# Impact of ultrasound in diagnosis of hydatidiform mole in early pregnancy: A protocol of systematic review

**DOI:** 10.1097/MD.0000000000022268

**Published:** 2020-10-09

**Authors:** Li Li, Cai-Yun An

**Affiliations:** a Department of Obstetrics and Gynecology, Yan’an Hospital of Traditional Chinese Medicine; b Department of Ultrasound Diagnosis, Yan’an People's Hospital, Yan’an, China.

**Keywords:** hydatidiform mole, impact, pregnancy, ultrasound

## Abstract

**Background:**

This study aims to assess current evidence of ultrasound in diagnosis of hydatidiform mole (HM) in early pregnancy (EP).

**Methods:**

This study will incorporate case-control study on investigating the impact of ultrasound in diagnosis of HM in EP. Potential articles will be retrieved in electronic databases of Cochrane Library, MEDLINE/PUBMED, EMBASE, PsycINFO, WANGFANG, and CNKI from inception to the present. Conference proceeding, website of clinical trial registry, and reference list of key articles will be examined for additional studies. Two independent researchers will scan and select studies, collect and manage data, and appraise methodological quality of all eligible studies. We will carry out summary effect size, statistical heterogeneity, synthesize, and analyze outcome data.

**Results:**

This study will summarize present evidence to assess the accuracy of ultrasound in diagnosis of HM in EP.

**Conclusion:**

This study may provide evidence for ultrasound in diagnosis of HM in EP, which may benefit both patients and clinicians.

**Study registration:**

INPLASY202080080.

## 1 Introduction

Hydatidiform mole (HM) is the most common type of gestational trophoblastic disease,^[1,2]^ which originates from the placenta and can metastasize.^[1,3]^ It is divided as complete and partial types.^[1]^ It has been estimated that the incidence of complete HM is about 1–3/1000 pregnancies, and the incidence of partial HM is about 3/1000 pregnancies.^[4,5]^ It usually occurs in extremely maternal age population, including females younger than 20 years old and older than 35 years old.^[6]^ It often manifests as vaginal bleeding, vomiting, no fetal heart, and human chorionic gonadotropin exceeding 80,000U/L.^[1,7]^ Although it is usually diagnosed benign, it has potential to become malignant and invasive.^[8,9]^ Thus, it is very important to detect this disorder at early stage.

Ultrasound is often utilized to detect HM in early pregnancy (EP).^[10–15]^ Although a growing number of studies report that ultrasound can accurately detect HM in the early stage of pregnant period,^[10–26]^ no systematic review has been conducted to appraise its diagnostic accuracy for HM. Therefore, in order to better understand its impact on HM, this systematic review aims to assess the accuracy of ultrasound in diagnosis of HM in EP.

## 2 Methods

### 2.1 Study registration

We have registered this study protocol through INPLASY202080080, and we have reported it in term of the guideline of Preferred Reporting Items for Systematic Reviews and Meta-Analysis Protocol statement.^[27]^

### 2.2 Eligibility criteria for study selection

#### 2.2.1 Type of studies

We will consider case-controlled studies (CCSs) for inclusion that reported the impact of ultrasound in diagnosis of HM in EP.

#### 2.2.2 Type of participants

We will consider female participants (more than 18 years old) with HM in EP in this study. No limitations were applied to race, country, and educational background.

#### 2.2.3 Type of index test

Index test: ultrasound was applied in diagnosis of HM in EP.

Reference test: patients were diagnosed by histological-proven HM in EP.

#### 2.2.4 Outcome measurements

Outcomes are sensitivity, specificity, mistake diagnostic rate, and omission diagnostic rate.

#### 2.2.5 Exclusion criteria

We will exclude animal study, nonclinical study, review, case report, and case series. In addition, we will not consider CCSs with sample size less than 10 patients.

### 2.3 Search strategy and information sources

This study will perform search strategy from literature sources and it will consist of 2 steps. First, a comprehensive search of potential studies will be carried out in Cochrane Library, MEDLINE/PUBMED, EMBASE, PsycINFO, WANGFANG, and CNKI from inception to the present. A sample of search strategy for Cochrane Library is presented in Table 1. We will modify similar search strategy for other electronic databases. Second, conference abstract, ongoing studies in clinical trial registry, and reference lists of essential studies will be identified for additional studies. No language and publication status limitations will be occupied in this study.

**Table 1:**
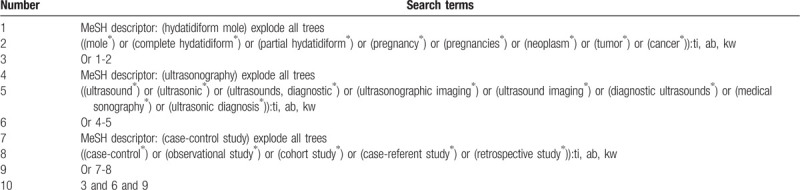
Search strategy of Cochrane Library.

### 2.4 Data collection and analysis

#### 2.4.1 Study selection

All searched citations will be exported into EndNote X7, and all duplicated records will be eliminated. Subsequently, 2 researchers will independently scan all titles and abstracts following the inclusion criteria. Then, full-text of selected articles will be read carefully against all eligibility criteria. We will exclude ineligible studies and record them with reasons. If any different views arise between both of them, we will seek a third researcher to solve them through discussion. The process of study selection will be demonstrated in a flow chart.

#### 2.4.2 Data collection and management

All essential data will be collected independently by 2 researchers using a developed and standardized sheet to document study information (eg, title, first author, year, study design, sample size, et al), patient characteristics (eg, age, gender, stage of tumors, et al), outcome measurements, index and reference tests, main findings, and other important information. If there are conflicts between 2 researchers, a third researcher will be invite to solve them, and a final decision will be reached. In addition, any lacking or missing information will be requested by obtaining original study authors via email or fax.

### 2.5 Quality assessment

Two researchers will independently appraise study quality of CCSs using Quality Assessment of Diagnostic Accuracy Studies tool-2.^[28]^ Any divergence will be resolved with the help of a third researcher through discussion or consultation.

### 2.6 Statistical analysis

Stata 12.0 software will be applied to conduct the statistical analysis. We will estimate effect size as descriptive statistics and 95% confidence intervals. Levels of heterogeneity will be identified using *I*^*2*^ statistic. *I*^*2*^ ≤ 50% means acceptable heterogeneity, and a fixed-effects model will be employed for data pooling; while *I*^*2*^ > 50% indicates obvious heterogeneity, and a bivariate random-effects model will be placed for data synthesis. If considerable heterogeneity is detected, we will conduct a subgroup analysis to find out its sources. We will carry out the pooled sensitivity, specificity, mistake diagnostic rate, omission diagnostic rate, and draw the hierarchical summary receiver operating characteristic curve to appraise diagnostic accuracy of ultrasound for HM in EP. If permits, we will pool the data and perform meta-analysis. If statistical synthesis is not possible, we will report its findings in forest plot and narrative description.

### 2.7 Subgroup analysis

We will conduct subgroup analysis in accordance with different study information, study characteristics, and index and reference tests.

### 2.8 Sensitivity analysis

We will perform sensitivity analysis to check stability of merged outcome results by removing low quality studies, or insufficient sample size.

### 2.9 Reporting bias

We will carry out funnel plot and associated regression tests^[29,30]^ to examine if there is any reporting bias.

### 2.10 Ethics and dissemination

This study will be conducted based on the published data, thus no ethic approval is required. It will be published on an associated peer-reviewed journal.

## 3 Discussion

Although numerous studies report the diagnosis of ultrasound for HM,^[10–26]^ no systematic review specifically investigate the accuracy of ultrasound in diagnosis of HM in EP. Thus, this systematic review will firstly explore the accuracy of ultrasound in detection of HM in EP by synthesizing effect estimates from all included CCSs. The results of this study may provide evidence for clinical practice. However, this study protocol may still have several limitations, including insufficient eligible CCSs, small sample size of included study, and poor methodological quality of included CCSs.

## Author contributions

**Conceptualization:** Li Li, Cai-Yun An.

**Data curation:** Li Li, Cai-Yun An.

**Formal analysis:** Li Li, Cai-Yun An.

**Investigation:** Cai-Yun An.

**Project administration:** Cai-Yun An.

**Resources:** Li Li.

**Software:** Li Li.

**Supervision:** Cai-Yun An.

**Validation:** Li Li, Cai-Yun An.

**Visualization:** Li Li, Cai-Yun An.

**Writing – original draft:** Li Li, Cai-Yun An.

**Writing – review & editing:** Li Li, Cai-Yun An.

## Abbreviations

Abbreviations: CCSs = case-control studies, CIs = confidence intervals, EP = early pregnancy, HM = hydatidiform mole.
